# Experiences of outreach workers in promoting smoking cessation to Bangladeshi and Pakistani men: longitudinal qualitative evaluation

**DOI:** 10.1186/1471-2458-11-452

**Published:** 2011-06-09

**Authors:** Rachna A Begh, Paul Aveyard, Penney Upton, Raj S Bhopal, Martin White, Amanda Amos, Robin J Prescott, Raman Bedi, Pelham M Barton, Monica Fletcher, Paramjit Gill, Qaim Zaidi, Aziz Sheikh

**Affiliations:** 1 UK Centre for Tobacco Control Studies, Primary Care Clinical Sciences, University of Birmingham, Birmingham, B15 2TT, UK; 2 Psychological Sciences, Institute of Health and Society, University of Worcester, Worcester, WR2 6AJ, UK; 3 Centre for Population Health Sciences, University of Edinburgh, Edinburgh, EH8 9AG, UK; 4 Fuse, UKCRC, Centre for Translational Research in Public Health, Institute of Health & Society, Newcastle University, NE2 4HH, UK; 5 UK Centre for Tobacco Control Studies, Centre for Population Health Sciences, University of Edinburgh, Edinburgh, EH8 9AG, UK; 6 International Centre for Child Oral Health, King's College London, London, WC2B 5RL, UK; 7 Health Economics, University of Birmingham, Birmingham, B15 2TT, UK; 8 Education for Health, Warwick, CV34 4AB, UK; 9 British Heart Foundation, London, W1H 6DH, UK; 10 Allergy & Respiratory Research Group, Centre for Population Health Sciences, University of Edinburgh, Edinburgh, EH8 9AG, UK; 11 CAPHRI, University of Maastricht, The Netherlands

## Abstract

**Background:**

Despite having high smoking rates, there have been few tailored cessation programmes for male Bangladeshi and Pakistani smokers in the UK. We report on a qualitative evaluation of a community-based, outreach worker delivered, intervention that aimed to increase uptake of NHS smoking cessation services and tailor services to meet the needs of Bangladeshi and Pakistani men.

**Methods:**

This was a longitudinal, qualitative study, nested within a phase II cluster randomised controlled trial of a complex intervention. We explored the perspectives and experiences of five outreach workers, two stop smoking service managers and a specialist stop smoking advisor. Data were collected through focus group discussions, weekly diaries, observations of management meetings, shadowing of outreach workers, and one-to-one interviews with outreach workers and their managers. Analysis was undertaken using a modified Framework approach.

**Results:**

Outreach workers promoted cessation services by word of mouth on the streets, in health service premises, in local businesses and at a wide range of community events. They emphasised the reasons for cessation, especially health effects, financial implications, and the impact of smoking on the family. Many smokers agreed to be referred to cessation services, but few attended, this in part being explained by concerns about the relative inflexibility of existing service provision. Although outreach workers successfully expanded service reach, they faced the challenges of perceived lack of awareness of the health risks associated with smoking in older smokers and apathy in younger smokers. These were compounded by perceptions of "lip service" being given to their role by community organisations and tensions both amongst the outreach workers and with the wider management team.

**Conclusions:**

Outreach workers expanded reach of the service through taking it to diverse locations of relevance to Pakistani and Bangladeshi communities. The optimum method of outreach to retain and treat Bangladeshi and Pakistani smokers effectively in cessation programmes needs further development.

## Background

There are marked ethnic and gender variations in smoking prevalence in the UK. In 2004, 40% of Bangladeshi men, 29% of Pakistani men and 24% of the general male population in England were smokers [1]. Stopping smoking is particularly important in Bangladeshi and Pakistani groups because their rates of heart disease, stroke and Type 2 diabetes are higher than in the rest of the population [2,3]. As part of a broad tobacco control strategy the UK has, uniquely in the world, established local NHS smoking cessation services which provide effective support for quitting [4]. These services have been effective in reaching disadvantaged groups including those from low socioeconomic groups [5]. However, uptake in South Asian groups (mainly Indian, Pakistani and Bangladeshi) in 2005 was half that of other groups [6]. This lower use of cessation services may reflect cultural differences in relation to awareness about and perceptions of such services, beliefs about the benefits of support and pharmacotherapy such as nicotine replacement therapy (NRT) in helping smokers quit, as well as the practicalities of service access [7-9]. Despite these concerns, no evaluated service models have been shown to overcome these barriers [10].

Improvements in service uptake and cessation rates among ethnic minority groups are likely to come from the development of culturally appropriate interventions [11]. One potentially promising approach is the use of lay outreach workers to promote smoking cessation and uptake of support services, but few studies have evaluated this approach. One randomised controlled trial in the US involved lay health workers delivering home-based smoking cessation programmes, tailored specifically to the cultural beliefs and practices of Latino smokers [12]. One week abstinence rates were twice as high in the intervention group (20.5%) compared to a helpline control group (8.7%, P < 0.005). A Cochrane review found that community lay health workers have been effective in primary care in promoting immunisation and improving outcomes for some infectious diseases [13]. In the UK, NHS cessation services have developed community outreach interventions with lay workers, but these have not been formally evaluated.

We conducted a 12-month pilot cluster randomised controlled trial of community outreach workers operating in Birmingham UK, which aimed to improve access to and the effectiveness of smoking cessation services for male adult Bangladeshi and Pakistani smokers [14], the majority of whom are of Muslim backgrounds and many of whom will therefore have a shared range of beliefs and experiences about smoking and smoking cessation [7,8]. We focused on men as smoking prevalence in these groups is much higher in men than women [1]. We randomised geographic communities to either control areas, where standard NHS stop smoking advisors (SSA) operated smoking cessation clinics, or intervention areas. Community outreach workers in the intervention areas referred smokers to existing clinics or provided cessation support themselves. The outcome evaluation, which will be reported elsewhere (Begh et al., 'Promoting smoking cessation in Pakistani and Bangladeshi men in the UK: pilot cluster randomised controlled trial of trained community outreach workers', submitted), found that the intervention increased the rate at which Pakistani and Bangladeshi men used cessation services, with a rate ratio of 1.32 (95% CI: 1.03-1.69). However, those who tried to quit in the intervention areas were somewhat less likely to succeed than those in the control areas, with an odds ratio of 0.86 (95% CI: 0.52-1.42). The net effect was a suggestive, but non-statistically significant increase in smoking cessation from 25 to 31/1000 smokers/year in the intervention areas, a rate ratio of 1.30 (95% CI: 0.82-2.06). However, as a pilot trial, the study was not powered to detect unequivocally such a difference.

It is important to evaluate the processes involved in complex interventions as it is the appreciation of these processes or mechanisms that offer the opportunity to develop potentially transferable insights and lessons [15,16]; the need for an in-depth appreciation of these process-related considerations is particularly important when undertaking formative work, which we hoped would inform our planned follow-on Phase III trial. Accounts of how community-based outreach workers promote smoking cessation are however uncommon [17]. We therefore conducted a qualitative process evaluation which aimed to help interpret the trial's findings and to understand more fully the approaches outreach workers and their management teams took when recruiting service users and supporting smoking cessation. The longitudinal dimension of this process evaluation was important to explore how their role and approach changed over time in response to initial difficulties. This paper draws on a range of complementary qualitative data to understand both individual and group related perspectives and experiences on how this complex intervention was delivered and how these changed over time with a view to teasing out implications for future initiatives, which aim to reach out to and support these populations in the context of promoting smoking cessation.

## Methods

### Design

Focus group discussions with the five community outreach workers were conducted at regular intervals during this 12 month trial, which was conducted in Heart of Birmingham Primary Care Trust (PCT) and Birmingham East and North PCT (HoB and BEN PCTs), Birmingham, UK. These focus groups were facilitated by one of the authors (RAB) and were supplemented with observation of management meetings, examination of outreach workers' weekly dairies, and shadowing of the outreach workers' activities. One-to-one exit interviews (with RAB) were conducted with outreach workers and three representatives from their management teams at the end of the study. Ethical approval was obtained from South Staffordshire NHS Research Ethics Committee.

### Participants

Five male community outreach workers (aged 24-49) participated in focus groups, interviews and observations. Four of the outreach workers (two Bangladeshi, two Pakistani) were recruited at the start of the study. Two outreach workers (one Bangladeshi, one Pakistani) were experienced stop smoking advisors. Outreach workers undertook detailed mapping of key resources that might support smoking cessation in their geographical areas and also completed two weeks of training in how to deliver behavioural support and medication management for smoking cessation, general health promotion, communication skills, and sessions on cultural diversity to understand the range of cultural-related beliefs, practices and experiences that may be of norms of Pakistani and Bangladeshi smokers; this training however also warned against the dangers of stereotyping on the basis of, for example, ethnic origin, language or age. The training involved role-playing a range of scenarios in the main relevant languages (i.e. Sylheti, Bengali, Mirpuri, Urdu, and English). All outreach workers were assessed as competent based on these role-plays by the end of training. The training was delivered by accredited NHS trainers and members of the research team. One Pakistani outreach worker resigned after six months and was replaced by a Pakistani stop smoking advisor who participated in the last focus group only. In addition, both local stop smoking service managers were interviewed. A stop smoking advisor specialising in ethnic minority health that helped manage the outreach workers during the final five months of the intervention was also interviewed. The characteristics of all participants are summarised in Table 1.

**Table 1:** Participant characteristics

Participant*	Sex	Ethnicity	Occupation
Rashed	Male	Bangladeshi	Outreach Worker
Tariq	Male	Bangladeshi	Outreach Worker
Wasim	Male	Pakistani	Outreach Worker
Samir	Male	Pakistani	Outreach Worker
Faheem	Male	Pakistani	Outreach Worker
M1	Male	White British	Local Stop Smoking Service Manager
M2	Female	White British	Local Stop Smoking Service Manager
M3	Female	Indian	Specialist Stop Smoking Advisor

*Pseudonyms

### Data generation

We adopted a mixed methods approach to data generation involving approaches to understand both individual and group perspectives. The data sources are summarised in Table 2.

**Table 2:** Data sources

Sources of data	Participants
Focus groups	Outreach workers

One-to-one exit interviews	Outreach workers, local stop smoking service managers, specialist stop smoking advisor

Management meetings	Outreach workers, local stop smoking service managers, specialist stop smoking advisor

Shadowing of outreach workers	Outreach workers

Outreach worker weekly diaries	Outreach workers

Focus groups have been widely used in process evaluations of health promotion interventions [18,19]. They are a useful tool for ascertaining the combined local perspectives of the implementers involved in delivering interventions [20]. The first focus group was carried out before the outreach workers began delivering the intervention to explore initial ideas about their role. Four further focus groups were held every two-three months. A topic guide was devised to explore the outreach workers' role, the approaches used to promote smoking cessation and the barriers encountered during implementation. This evolved as new themes emerged. Ten management meetings between the stop smoking service managers and the outreach workers were observed to understand team dynamics and how the approach unfolded. Focus group and management group discussions lasted 1-2 hours.

In addition, the study researcher (RAB) shadowed each outreach worker for several hours and made detailed notes to understand the nature of the work. The outreach workers kept weekly diaries, which encouraged recording structured and unstructured information about the details of the work undertaken including the type, location and duration of activities and their own observations on the value of these activities. The diaries were collected monthly by the management team and copies were given to the study team. Finally, individual exit interviews were carried out with the outreach workers, the stop smoking service managers and the specialist stop smoking advisor at the end of the study. Topic guides were used to explore the rewards and challenges of the outreach worker role, their overall performance and impact on the public, and reflections on working in the team.

### Data handling and analysis

The focus group discussions, interviews and all but one management meeting were audio-recorded. Twenty-one audio recordings were transcribed and analysed. Diaries were collated and thematically summarised, together with the researcher's notes from the observations of the outreach workers. Given the range and volume of data, we undertook analysis using a modified Framework approach, which involved five main stages: familiarisation, identifying a thematic framework, indexing, charting, mapping and interpretation [21]. Familiarisation with the data was an iterative process that occurred throughout data collection. Issues that emerged from the data were indexed and collated to form broad categories within a loose thematic framework. These were examined and emergent themes identified in regular discussions of the qualitative sub-group (RAB, PA, AA and AS) with emerging ideas incorporated into subsequent rounds of data collection. The range of data sources allowed identification of both commonly held and contrasting perspectives. We searched for disconfirming data and continued data analysis until no major new themes emerged in relation to the main questions of interest. We finally examined the relationship between quotes and linkages between the data as a whole. The major themes and sub-themes are summarised in Table 3.

**Table 3:** Major themes and sub-themes

Major theme	Sub-theme
	Role development
Working with target group	Encouraging cessation
	Encouraging use of pharmacological therapies
	Difficulties in encouraging cessation and use of stop smoking services

Working with local service partners	Initial outreach approach
	Challenges with initial outreach approach

Working with local relevant businesses, organisations and	Using resources within the community
institutions	

Working with fellow members of the outreach team and managers	

## Results

We present first the views and experiences of the outreach workers on working with service users, local service partners and local South Asian businesses and organisations. We also present their views on working with fellow members of the outreach team and the views of their local stop smoking service management team on their overall performance. Supporting illustrative quotes are presented with pseudonyms to preserve anonymity.

### Perspectives of outreach workers

#### Working with the target group

##### Role development

The outreach workers initially shared the view that their primary function was to increase awareness in Bangladeshi and Pakistani smokers about local stop smoking services and encourage use among those wishing to quit smoking. They anticipated that their working methods would develop with experience, and through trial and error.

"I think until you go out there and try some of these methods out you wont really know which one will be most effective, which one will be the least effective and I think that's what the whole of this project is really about having a bit of trial and error... And that's what's within the flexibility of outreach is the ability to change and swap and use that to explore different avenues." (Samir, FG1)

The importance of building relationships with individuals was frequently mentioned. Outreach workers agreed that gaining the trust and respect of the community was essential to relationship building.

"It's about building the relationship, building the rapport and as soon as they see that okay this person is here for my benefit then they will start opening up. This is how our culture works." (Samir, FG1)

However this was sometimes a challenge during outreach on the streets, as some people were suspicious of their motives. In an attempt to dispel suspicions, they sometimes adapted their approach depending on the age and language spoken by the individual, for example, using "street language" and "buzz words" with younger smokers and addressing older smokers as "uncle" to gain respect. The NHS was frequently mentioned as it is trusted by most Bangladeshi and Pakistani people.

Outreach workers believed that developing their local profile was essential to forming relationships with the community. This involved using publicity, for example, handing out business cards and by word of mouth. They approached Pakistani and Bangladeshi people opportunistically on the streets around shopping areas. They believed that their repeated presence in the community would, over time, help develop trust. This appeared to work, because some smokers who were initially unwilling to quit subsequently approached the workers for advice on quitting.

"Signed up client whom I had seen several times on Coventry Road and is now ready to try and quit." (Rashed, diary entry)

Outreach workers believed that their acceptance by the community was because they shared the same ethnicity and/or religion. During observations, exchanges with potential clients were often initiated through outreach workers expressing their identity. Initial exchanges often involved shaking hands and using the religious expression "*Assalamu alaikum" *("Peace be with you"); thus immediately expressing their religious identity. Additionally, conversing in Bengali, Urdu or Mirpuri identified the outreach worker as of Bangladeshi or Pakistani origin. Outreach workers emphasised that they were there to specifically help Pakistani and Bangladeshis. They believed that such displays of communality offered reassurance, facilitated communication, and generated respect for them and their role.

"It helps to relate, for them to relate to you, doesn't it? Because if they feel you're from the same country or if you're not from the same country it's the same religion, they can relate to... they feel they can relate to you. I think that's why they ask these questions: where are you from, are you Muslim... or whatever." (Tariq, FG4)

##### Encouraging cessation

Outreach workers believed that concerns about the likely health impact of smoking were a key lever to enhancing smokers' motivation to stop. Messages were tailored to age and interests. For young men, particularly those interested in physical activity, the benefits of having a healthier lifestyle were emphasised. This was reinforced by offers of free use of local city council gyms if they signed up to the stop smoking programme.

"For the younger generation we would highlight the things on terms of, for example, sport, activities, holidays, things that they could get, buy." (Wasim, FG4)

In contrast older men were reported as being unaware of the major health consequences of smoking except lung cancer. They therefore frequently discussed the benefits of quitting for short-term and long-term health.

"Just simple things, like circulation, when you explain to them about how it effects the circulation, they say 'oh yeah, that's why in winter my hands and feet are always cold'. And we explain that, well, if you did stop that would improve within 20 minutes. And, like, they're quite surprised." (Rashed, exit interview)

Smokers were also advised of the financial benefits of stopping smoking, for themselves and their family. The effects of smoking on the family were frequently discussed with smokers and also the wider community (typically non-smoking women) at various community events. Most thought that this was useful, but one outreach worker doubted this. He believed that coercion by family members was not always helpful as it could attract smokers who were not fully committed to quitting and would drop out.

"If it was a man that was supposed to have come for an appointment or he has started a programme, he's not coming here because he really wants to stop, its because he's been coerced by his wife or his mom or he's being pushed into it and he's not ready to stop, that seems to be the main problem. But where they have come for themselves, the drop-out rates are very low." (Tariq, exit interview)

In contrast, another outreach worker argued that targeting families could reduce passive smoking, citing several cases where family members had banned smoking in their home.

Outreach workers often used "shock" tactics through pictorial images and visual props. They developed culturally specific posters and leaflets and used feedback to refine these before distributing them to the public, community networks, and pharmacists.

"The fact that they're smoking the rat poison stuff that they've got in their shops, they can't believe they've got these items in them and that's what's been working for me" (Samir, MM1).

##### Encouraging use of pharmacological therapies

Bangladeshi and Pakistani smokers were reported to be lacking in knowledge about and belief in pharmacological aids to quitting. Although many smokers knew of and had used nicotine replacement therapy (NRT), they had done so inappropriately. Some thought that using NRT would make them want to stop smoking and that, if it had not done so, it did not work.

"What happened was originally they would have come back to us and said, 'Oh, I've tried to stop smoking before with this patch and it didn't work for me'. But when you explain to them or you ask them as to how they used it, that's when you find out they haven't been doing it properly or they borrowed the patch from somebody else or they've got one from their friends or cousins or family or etc as a one-off." (Wasim, FG5)

Outreach workers attempted to correct these misconceptions, making smokers more aware of the purpose and benefits of using NRT - for example, referring to NRT as a form of medication in order to emphasise the importance of completing a full course of treatment.

"Some of the people didn't see it as a medication until we spoke to them basically. So a lot of people don't think it's an option, they think it's just like a gimmick." (Wasim, exit interview)

During Ramadan, when Muslims fast during daylight hours and cannot eat, drink or smoke, advice about NRT was tailored specifically to the individual's religious beliefs. There were, for example, differences in views about the religious legality of using NRT patches during daylight hours. Where people had reservations they were advised to use patches upon completion of a day's fast.

##### Difficulties in encouraging cessation and use of stop smoking services

The most common challenge cited was Bangladeshi and Pakistani smokers' lack of interest in stopping smoking. Young people were particularly unwilling to quit, even though they knew more of the health risks than older smokers, as they enjoyed smoking.

Both Bangladeshi outreach workers stated that, compared with Pakistani smokers, Bangladeshi smokers were harder to motivate to access the services and were less likely to want to quit. However, if they made the commitment to change, Bangladeshi clients were perceived to be more committed to the stop smoking programme than Pakistani clients.

"The thing is with the Pakistani community, if they're easier to convince to try it, they might give it a go or they might not be fully into it, rather when... you know like I said its difficult to motivate the Bangladeshi community but eventually when they do come through it seems to be like, they're really like committed." (Rashed, exit interview)

Addressing smokers' negative attitudes towards seeking and receiving help from the stop smoking services was also a challenge. Some questioned the value of the support and advice offered by the cessation service, particularly if they had never experienced support previously. Others believed that they could quit without assistance.

"...another thing is they have this attitude that the support, I mean 'What's that going to do for me?'" (Rashed, exit interview)

Outreach workers also found that smokers who lapsed during their quit attempt were reluctant to come back for their weekly session, believing this disqualified them or because they felt ashamed.

### Working with local service partners

#### Initial outreach approach

At the start of the intervention, smokers were referred to local cessation services provided by pharmacies, GP practices and drop-in clinics. Bangladeshi and Pakistani smokers were thought to be unaware of these services.

"We're highlighting the services that they didn't even know about, especially where you've got cases where next neighbours didn't know that the pharmacist next door, the business next door, provides that sort of service." (Wasim, FG4)

Initial outreach activities therefore involved collaborations with existing service providers to increase awareness. Smokers completed a referral form if they were interested in attending their local SSS and outreach workers telephoned them fortnightly to establish whether they had attended and to reinforce the behavioural support given by the SSA if they had. In addition, outreach workers visited pharmacies and GP practices weekly to determine whether referred smokers had attended.

Outreach workers offered to provide interpreting services for behavioural support for cessation in pharmacies and GP practices. Promotional events were arranged in GP practices to attract smokers. However, working with GP practices proved difficult to arrange because practice managers were often unavailable.

Pharmacies were the main venue for smoking cessation support of Pakistani and Bangladeshi smokers. However, initial offers by the outreach workers to be present in the pharmacies to help engage with Pakistani and Bangladeshi smokers was met with resistance from some pharmacists, who perceived them as a threat to their business. (Pharmacists are paid on a contract to provide cessation services).

Working with other agencies involved in public health proved to be easier. These included Neighbourhood Development Officers and Healthy Heart workers. Being present at cardiovascular disease clinics complemented outreach activity because the clinics attracted Bangladeshi and Pakistani people. They found that people expressed more interest in quitting when smoking cessation was linked with other health issues.

#### Challenges with initial outreach approach

Half way through the intervention period it became apparent that most of the smokers who accepted a referral did not access the cessation service. Several reasons for this were identified, relating to service provision and smokers' issues.

The existing types of cessation service provision was felt by the outreach workers to create access barriers, being at fixed times in the day or not available when required, for example, because the pharmacist was too busy.

"In some cases when they do turn up, the timing for which the pharmacist can see them is not suitable to them...either because they've gone to the pharmacist on the time they made an appointment of, or entered the chemist at a time that it's busiest time as well, so the pharmacist doesn't have the time to deal with them. And when they do ask them to come back again, the pharmacists do come back to us and tell us that they haven't turned up although they've given them an appointment." (Wasim, FG4)

They described the service in many pharmacies as too formal and impersonal. Smokers were typically confused over pharmacy appointment systems and when consultations were arranged, they were reportedly often hurried. Older smokers, who often had difficulties speaking and understanding English, were said to be reluctant to use existing services because of language barriers. Although the outreach workers offered to accompany smokers to their local service provider and offered language interpretation, there was little uptake.

"There is a lot of motivated people out there who do want to quit and language is a problem for a lot of them because a lot of them are over the age of 35 and their English is pretty poor... they're wary of going somewhere and not being able to speak English, understand even." (Tariq, exit interview)

Outreach workers also thought that some smokers agreed to referral out of politeness or became motivated to quit when discussing this with them but this dissipated later. Two outreach workers also thought that some people did not want to be referred on to a "third party" and that it was important that the relationship developed during initial interactions was built on to help people to quit.

"When we're out on the streets or wherever when we're talking to somebody, people probably feel more comfortable anyway because we've already broken the ice with them by speaking to them and informing them and plus, at the same time they'll already know us by that time anyway, they're not just going on to a third party place where they're standing around." (Wasim, MM7)

Outreach workers also felt that young people feared disclosure of their (hidden) smoking status since their families and other community members used the pharmacy or GP practice where the cessation service was located. Also many staff there were of the same ethnic/religious background and hence known to the family.

These reasons informed the change of approach half way through the intervention period. It was agreed that outreach workers, rather than referring to existing services, would set up their own clinics. These were one-to-one rather than group consultations, to reduce concerns over disclosure.

### Working with local relevant businesses, organisations and institutions

#### Using resources within the community

Collaborating with local businesses and organisations was seen as an additional way of accessing Bangladeshis and Pakistanis. Outreach workers approached money transfer businesses, taxi firms, supermarkets, fast food outlets and butchers shops. Initially local business people often had misconceptions, thinking that outreach workers were "enforcement officers" for the smoke-free legislation or from the "Department of Immigration". As when working with prospective clients, dressing casually rather than formally helped combat this impression.

"At the beginning we had a problem when we used to go round into supermarkets, restaurants etc, because they had this fear factor that we were coming from a different Government body apart from health, like they were scared like you know, they thought we were like immigration or something like that, but I think, it's like what I initially said it was like the way we were dressed, I suppose too uniformed, you know with a shirt and tie etc...but then when we changed our dress and the way we used to approach them, they were more comfortable to talk to us." (Wasim, exit interview)

Staff in these businesses were often interested in stopping smoking. It was hoped staff who enrolled in the stop smoking programme would pass on information about their experiences to customers which would increase customers' interest in accessing these services.

"So basically we get a lot of support from the taxi base owners, which is the first thing about it, and they're passing on the word. I mean we're giving out cards, but do you know what they've also been supportive in, is that they've took some of our leaflets, kept them in their taxi cabs and they've given them to one or two regular passengers that they know. So it's not only like hitting the taxi drivers, we've hit some of their passengers." (Wasim, FG3)

Taxi and bus drivers were often unable to be referred to an existing cessation service because of their shift patterns. So where there was sufficient interest, the outreach workers established their own clinics within taxi bases and bus depots.

Sports centres, adult education training centres, libraries and mosques were also approached as venues for holding their own drop-in clinics. Several barriers were encountered in developing such links and new services. While it was often difficult to gain access to mosques, one outreach worker, who was known to the committee members of a mosque in his area, was able to use this link to gain permission to carry out outreach work within the mosque. Holding clinics inside mosques proved largely ineffective because smokers feared being seen by family members while attending a cessation service. Promotions in mosques were therefore used to advertise clinics away from the mosques, for example, by the Imam to members of the congregation during the *'Khutbah' *(sermon) of the Friday congregational prayer.

Some outreach workers expressed frustration with working with South Asian businesses and community organisations. One outreach worker stated that although community organisations initially seemed interested in working with him, this was "lip service". Financial incentives were seen as important to entice some of these community organisations and businesses to work with them.

"What they were interested in is money more than anything else, and they'd be happy to work with the PCT [Primary Care Trust] as long as the PCT were paying them. And if the PCT does not want to pay them they're not going to work with them at all, they're never going to get any help from them at all." (Tariq, exit interview)

Restaurant owners were reluctant to declare that their staff smoked or to admit that they knew of people interested in stopping smoking because they allowed (illegal) smoking on the premises. They would also not let their staff go to clinics held in other restaurants because of fear that staff would be "poached" by competitors.

### Working with fellow members of the outreach team and managers

Outreach work was affected by difficulties in the team. Two particular issues were highlighted. One related to perceptions about the extent to which all the team members were pulling their weight, which was linked to absenteeism. The second issue concerned conflicting views about approaches to outreach work. In particular, whether street outreach work was necessary when one had local knowledge and therefore using personal community contacts was sufficient. While difficulties were apparent in one pairing, the managers felt that the other pairing was "hard working" and "very personable". They were thought to have integrated well with other health professionals, had raised awareness about cessation services and had "a presence" in the community. However, the problems with the other pairing led to disharmony within the team which was thought to have impacted on the team's performance.

Tensions were also expressed by outreach workers and managers about the clarity of the management structure, the responsibilities and challenges inherent in monitoring and supervising a novel intervention which by its nature was flexible, and differing expectations about performance and outcomes. For example, managers questioned whether the outreach workers were given too much flexibility in their role, as they felt unable to fully trust them to work without supervision.

"I'm not sure they were experienced enough to be relied upon to go out and actually manage their own workload and be expected to work off their own initiative. And also be trusted to work on their own unsupervised. And I think to some extent all of them kind of went off the rails a little bit and kind of took advantage of that kind of freedom and flexibility that post kind of offered. And that is not necessarily a criticism of them, it's a criticism of how they were managed really which wasn't best practice." (M1)

One manager felt disappointed and frustrated with the overall performance of the outreach workers, while two others felt that progress had been as expected. However while all managers had expected the outreach workers to develop their role and lead the project, most ideas were conceived by the management team.

"I think they did work well but I still felt that they could have done a bit more...I expected them to use a bit more of their initiative to go out and say 'Right, we're going to do a promotion, we're going to organise a promotion, this is what we're going to do' but no they were still waiting for us to, you know for ideas, hand them out, 'try this, or why don't you do this' or something." (M3)

One manager felt that many of the initiatives that were developed were often too short, with insufficient time to evaluate and draw lessons. On reflection, it was felt that perhaps insufficient start-up time was given at the beginning of the intervention for the outreach workers to develop the necessary skills for their role.

"...there was no time to really settle in, they had to hit the ground running. And I think that's something that everybody failed to take into account, that these guys would take so long to get up a head of steam..." (M1)

All outreach workers felt that the targets set by the management team at the start of the intervention were unrealistically high. They also expressed disappointment about the initial outreach approach which was not what some had expected. Being trained to deliver cessation support, some outreach workers felt that their skills were under-utilised by only referring smokers to existing services. This was compounded by the inflexibility of existing services, the low attendance rate, and the poor service experience reported to them by some clients. They believed that setting up their own clinics had been more successful in encouraging people to use the services and increasing adherence, but this development had been held back by being involved in a research project.

"I didn't realise because of the research that we were just going to refer people on to existing services. And again I don't think, the university or the different bodies involved didn't realise the effect that the existing services would actually have on the community there." (Rashed, exit interview)

However, the managers felt that most of the outreach workers lacked motivation towards the end of the intervention as they felt they had exhausted possibilities and started to look for new jobs and this was likely to have impacted on their effectiveness.

"...by the time they'd got up a head of steam, had some idea about what they were doing, they didn't have much left on their contracts and they weren't that motivated to bother because they were already thinking about their next job." (M1)

## Discussion

### Summary of main findings

The findings of this qualitative process evaluation highlight the importance of developing culturally appropriate methods to increase knowledge of and access to NHS smoking cessation services in Pakistani and Bangladeshi communities. They also highlight the difficulties of, and importance of allocating sufficient time for, developing a novel intervention in the context of a trial.

The study confirmed previous findings that many Pakistani and Bangladeshi smokers lack awareness of NHS smoking cessation services and their value [9]. Community-based outreach workers successfully addressed this by promoting cessation services through word of mouth on the streets, at community events, in businesses and mosques. They were able to communicate reasons for cessation to smokers and refer a substantial number for behavioural support and medication. However, our findings suggest that this was a frustrating process for both the workers, who felt deskilled, and for the management because the numbers referred to and, in particular, attending the cessation services fell far short of expectations. Consistent with previous findings [9], barriers to accessing the services arose from the inflexibility of existing services, smokers' feelings of mistrust towards providers and language barriers. These reasons led to a change of approach. Outreach workers started to provide their own clinics and although this was perceived by them to be a more successful and fulfilling approach, the number of smokers reached this way was too few to have a substantial impact on the number of Pakistani and Bangladeshi smokers stopping smoking in the community (Begh et al., submitted). Overall, service users in intervention areas were less likely to succeed in stopping smoking than those in the control areas. Our findings here suggest that the outreach approach may have enrolled people who were somewhat less motivated to stop smoking to use the service. Outreach workers often encountered people who generally lacked interest in stopping smoking and thought that some people signed up to the programme out of politeness.

The managers had initially high and perhaps unrealistic expectations for the intervention, which soon turned to disappointment. Although the outreach workers referred smokers, relatively few attended services. The outreach workers were wary of being unable to meet their targets, but they did not feel able to voice their concerns because of team dynamics. The management team also became dissatisfied because they believed that outreach workers should be developing 'bottom up' approaches to improving service use, but typically suggestions came from the management. These findings replicate other evaluations, where differences in expectations between management and workers led to problems in delivery [22,23].

Pharmacists were seen as key stop smoking service providers for this project because many of them were Pakistani Muslims and thus presumed to have some understanding of issues facing Pakistani and Bangladeshi smokers. It proved difficult to engage pharmacists in improving the service they offered these smokers. Pharmacists had a strong sense that their service was theirs and not much sense that it was part of a broader NHS service. Outreach workers were unable to break through this and they were not trained to help other health professionals in change management. Outreach workers had concerns about the quality and availability of these services, but felt unable to change this. They believed that the clinics which they set up in the later part of the intervention were more successful than existing cessation clinics in retaining smokers, but the empirical evidence suggested that, if anything, the reverse was true (Begh et al., submitted).

### Strengths and limitations

The study has several strengths. Qualitative evaluations of community-based interventions for improving smoking cessation rates are rare. By including a detailed qualitative evaluation, we were able to illuminate some reasons why the results were less encouraging than those originally envisaged. The longitudinal qualitative approach allowed us to explore how the outreach workers' roles and methods developed over time. We were also able to triangulate our findings using a variety of data sources (for example, focus groups, observations and interviews) to capture the diversity of perceptions and complexity of intervention activities.

Some aspects of data collection were less satisfactory than others, however. We asked the outreach workers to keep a diary of their activities, but they did not do this reliably. Most outreach workers recorded their daily activities retrospectively, therefore some information on the type and duration of activities was inaccurately recorded or missing. We also had concerns that the close relationship between the managers and the research team meant that the outreach workers might not always have been candid in focus groups and management meetings where they discussed their work. This might have been reinforced in earlier focus groups by the most vocal outreach worker, who gave answers that reflected his training and initial optimism surrounding the project. However, as the study progressed, the outreach workers appeared to become much more open. By including in-depth individual interviews, we were able to explore issues around the outreach workers' practices and team dynamics that they may not have felt comfortable disclosing in front of their managers or fellow team members. Another limitation of the study is that we did not collect data directly from Pakistani and Bangladeshi people that the outreach workers encountered. The findings instead relate to the outreach workers' perceptions on what they believed were barriers to smoking cessation for Pakistani and Bangladeshi smokers. However, this study confirms much of what has been found in previous research on Pakistani and Bangladeshi smokers' views on smoking cessation [9].

The Medical Research Council Framework for Development and Evaluation of Complex Interventions provides a guide to enable researchers and practitioners to develop interventions and assess the process of implementing them to ensure they are working as intended prior to a definitive trial [24-26]. Although different models of outreach work were attempted in this project, the one year intervention period meant that the project ended before a settled and effective intervention was developed. Sufficient time should have been allocated towards developing and refining the intervention with the outreach workers, prior to the start of the intervention period.

### Implications

This project was partly founded on research that Pakistani and Bangladeshis face specific cultural barriers in cessation [7-9]. One specific cultural issue related to the use of medication during Ramadan, which could constrain the choice of smoking cessation pharmacotherapy [27]. However, outreach workers identified the main reasons for cessation as health, money, and family, feelings shared by the general population [28-30]. The main misconceptions were about the value of behavioural support and NRT, which are shared by smokers in the general population [31,32]. In data not presented here, pharmacists (nearly all of Pakistani origin) reported giving the same advice to Pakistani and Bangladeshi smokers as to smokers from other cultural groups. However, these findings do not argue against outreach as such. Though the drivers of cessation and barriers to effective use of treatments to enhance success might be common to all groups of smokers, it is possible that the most effective way to address these issues in particular subgroups is by tailored approaches and the data suggest that the tailored approach used here was successful in raising the rate of use of cessation services.

Following this project, two outreach workers continued working in the stop smoking services; one as a stop smoking advisor in HoB and another as a black and minority ethnic specialist advisor in BEN. Further development and testing of outreach interventions to reach a settled model is required prior to moving to a phase III trial.

## Conclusions

Outreach workers successfully expanded the reach of NHS stop smoking services through innovative ways of working. The most consistent method used was service promotion by word of mouth, on the streets, in health service premises, and at community events. Outreach workers did this by emphasising common reasons for cessation (such as health, expense, and families), and by addressing common misperceptions of the value of behavioural support and pharmacotherapy for smoking cessation. Delivering this promotional work was successful, though somewhat unrewarding, perhaps because it fell short of arguably unrealistic expectations. Trials of outreach interventions should be of sufficient duration to allow embedding of new models of service delivery. Others developing outreach work for minority populations should consider these experiences in tackling these complex issues.

### Ethical approval

This study has been approved by South Staffordshire Local Research Ethics Committee.

## Abbreviations

BEN: Birmingham East and North Primary Care Trust; HoBt: Heart of Birmingham Teaching Primary Care Trust; NRT: Nicotine Replacement Therapy; PCTs: Primary Care Trusts; SSA: Stop Smoking Advisor.

## Competing interests

PA has done consultancy work for Pfizer, McNeil, and Xenva (now Celtic) Biotechnology with regard to smoking cessation. All other authors declare that they have no competing interests.

## Authors' contributions

AS and RSB conceived this study and together led the bid to secure funding for this work and manage the project. All authors contributed to the design of the study and oversaw its implementation. RAB was responsible for conducting the interviews, observations and focus groups and managing data collection. RAB, AA, PA and AS contributed to design of the research materials, data analysis, data validation and drafted the manuscript. PU, RSB, PG, MW, RJP, RB, PMB, QZ and MF all commented on draft versions of the manuscript. All authors read and approved the final version of the manuscript prior to submission.

## Pre-publication history

The pre-publication history for this paper can be accessed here:

http://www.biomedcentral.com/1471-2458/11/452/prepub
